# Curve-centered plaques raise the risk of peri-operative neurological and cardiovascular complications during angioplasty and stenting for severe carotid stenosis

**DOI:** 10.3389/fneur.2024.1455135

**Published:** 2024-10-07

**Authors:** Chul-Hoo Kang, Jong-Kook Rhim, Hong Jun Kim, Jay Chol Choi, Joong-Goo Kim

**Affiliations:** ^1^Department of Neurology, Jeju National University Hospital, Jeju National University School of Medicine, Jeju, Republic of Korea; ^2^Department of Neurosurgery, Jeju National University Hospital, Jeju National University School of Medicine, Jeju, Republic of Korea

**Keywords:** carotid stenosis, carotid stenting, stroke, complication, carotid angiography

## Abstract

**Introduction:**

Carotid artery stenting is an alternative interventional treatment to carotid endarterectomy. However, preprocedural considerations and anatomical risk factor analyses for carotid artery stenting are currently insufficient. Therefore, we investigated the high-risk anatomical appearance of carotid artery stenting from the neurointerventionist perspective to predict periprocedural complications.

**Materials and methods:**

We retrospectively reviewed patients with carotid stenosis who underwent carotid artery stenting at a comprehensive stroke center between January 2012 and December 2021. We compared the demographic characteristics, medical history, and anatomical appearance of the stenotic segment in patients with and without complications.

**Results:**

We analyzed a total of 148 patients (64 women [43.2%]; median age, 73.0 [interquartile range, 65.5–79.0]). Complications occurred in 39 of the 148 patients, primarily minor and transient. Of baseline or procedural characteristics, a high initial National Institutes of Health Stroke Scale score (*p* = 0.04), symptomatic stenosis (*p* = 0.01), and curve-centered plaque of the proximal ICA (*p* = 0.01) were significantly associated with carotid artery stenting complications in unadjusted analysis. Curve-centered plaque remained an independent risk factor for carotid artery stenting complications after adjustment (odds ratio 2.23[1.02–4.88], *p* = 0.04).

**Conclusion:**

High-risk vascular anatomical features, such as curve-centered plaque, are associated with a high frequency of periprocedural complications of carotid artery stenting. Tailored patient selection for carotid stenosis is crucial to prevent complications. Patients with curve-centered plaque should consider alternative treatment options such as carotid endarterectomy to achieve optimal clinical results.

## Introduction

Carotid stenosis is a significant cause of ischemic stroke (1). Carotid endarterectomy (CEA) and carotid artery stenting (CAS) are widely performed to prevent ischemic stroke in patients with carotid stenosis (2). Many studies have reported the effectiveness and safety of CAS (3, 4). CAS offers the advantages of being less invasive, reduced potential for postoperative wound complications, reduced postoperative pain, and a short length of hospital stay. Therefore, the frequency of CAS has recently increased (5, 6).

Nevertheless, tortuosity around the targeted neck vessels, which is relatively common in the older population (7), could affect the clinical outcomes of CAS (8, 9). Most periprocedural complications occur during the delivery of the devices, such as microwires, embolic protection devices (EPDs), or carotid stent systems to the stenotic segment (10). Thus, risk evaluation of high-risk anatomical appearances before CAS is crucial for optimal clinical outcomes.

During carotid artery stenting, we experienced that the stent or balloon often got caught in the plaque when the plaque was located in the center if the curved proximal ICA. A curve-centered plaque (CCP) is a large plaque associated with stenosis located within the severely tortuous carotid artery, which is the lesion target of the stenting procedure. Severe stenosis with a large plaque in a severely curved vessel can pose a substantial challenge in CAS (9, 10). Few studies have investigated the impact of a high-risk anatomical appearance with extracranial ICA tortuosity on CAS (9, 10).

This study aimed to analyze the association between high-risk anatomical appearances of the carotid artery, such as CCP, and periprocedural complications of CAS. We evaluated only neurological and cardiovascular complications.

## Methods

### Study population

**Figure 1:**
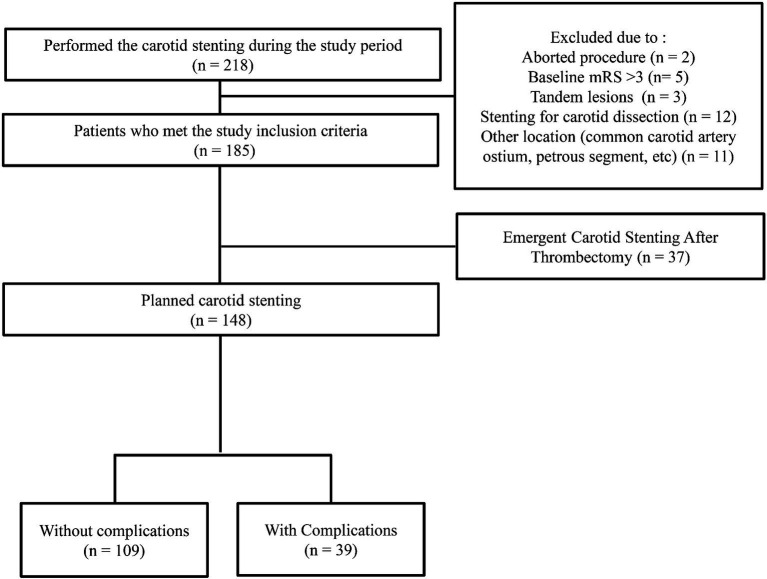
Flowchart of the patients.

Consecutive carotid stenosis patients who underwent endovascular balloon angioplasty and stent placement at a comprehensive stroke center between January 1, 2012, and December 31, 2021, were retrospectively selected from a prospective neuro-intervention database and stroke registry. The local institutional review board approved this study, and the need for written informed consent was waived because of the study’s retrospective nature. According to our management protocol, CAS was considered in all patients presumed to require revascularization. However, CAS was not considered for patients who did not have procedural equipoise or vasculitis or those who underwent dissection and iatrogenic occlusion (e.g., surgery or endovascular treatment). CAS was also not performed when patients or their proxies did not provide consent or when a medical condition contraindicated CAS. All procedures were performed when severe (>70%) underlying proximal carotid artery atherosclerotic stenosis was observed according to the NASCET criteria on DSA. Additionally, duplex procedures confirmed the detection of a peak systolic velocity >230 cm/s before the duplex ultrasound (DUS). CAS for asymptomatic carotid stenosis was performed for progressive stenosis on follow-up DUS, severe stenosis with ulceration, and contralateral carotid occlusion. Patients with a modified Rankin Scale of 3 or less who underwent CAS for atherosclerotic stenosis of the proximal ICA were included in the analysis. Patients with tandem lesions, those who underwent stenting at locations other than the proximal ICA, and those with emergent CAS were excluded. Depending on periprocedural events and long-term follow-up, patients were classified into the complication or non-complication group (Figure 1).

### Periprocedural antiplatelet medication

Dual antiplatelet therapy (DAPT) with aspirin and clopidogrel was generally administered to treat CAS. The patients received 100 mg aspirin and 75 mg clopidogrel daily for at least 14 days before the procedure. DAPT was administered upon admission to patients who had undergone acute ischemic stroke. If bleeding complications were absent, DAPT was continued for at least 6 months postoperatively, and single antiplatelet therapy was continued as life-long treatment.

### Clinical assessment and data acquisition

Patient data were retrospectively retrieved from a prospective database at our institute. The factors for comparison between the two groups were extracted from electronic medical records and included demographic characteristics (age, sex, and cerebrovascular risk factors), clinical characteristics (procedure-related information and modified Rankin scores at baseline and discharge), and radiographic and angiographic characteristics (complications, nature of stenosis, and devices).

### Intervention procedures

All endovascular treatments were performed by three neurointerventionalists at our institution who treated all patients with carotid stenosis. Except on emergency cases, CAS was performed in two stages after confirming the stenotic lesion and its characteristics through digital subtraction angiography. All procedures were performed via the percutaneous transfemoral route under local anesthesia. After placement of the sheath introducer, heparin was administered intravenously to maintain an activated clotting time >2-fold above normal. EPDs were used in all the patients. Spider FX (Medtronic, Minneapolis, Minnesota, United States) or Emboshield Nav6 (Abbott Vascular, Abbott Park, Illinois, United States) was mainly used as EPDs. Rarely, the Mo.Ma Ultra Proximal Cerebral Protection Device (Medtronic, Minneapolis, Minnesota, United States) was used at the discretion of the neurointerventionists. Typically, a 6F Shuttle (Cook Medical Inc., Bloomington, Indiana, United States) or NeuroMax (Penumbra Inc., Alameda, California, United States) was employed for the procedure. Predilatation was generally performed with a 3.0–4.0 mm diameter angioplasty balloon, and postdilatation was performed with a 4.0–7.0 mm diameter angioplasty balloon. A neurointerventionist determined the diameters of the balloon catheters. The stent was deployed in all cases, and the type of stent was determined at the discretion of the neurointerventionists. After dilatation, conventional angiography was repeated to evaluate periprocedural complications, including in-stent thrombosis or flow limitations. Finally, intracranial angiography was performed to confirm distal thrombus embolization.

### Imaging protocol

The final diagnosis of severe carotid artery stenosis was based on DUS, with peak systolic velocity and B-mode plaque images. All patients underwent conventional CT or MRI, including contrast-enhanced angiography of the carotid vessels. Two neuroradiologists who were blinded to the available clinical data independently assessed all images. Any discrepancies were resolved by consensus.

### Lesion characteristics

**Figure 2:**
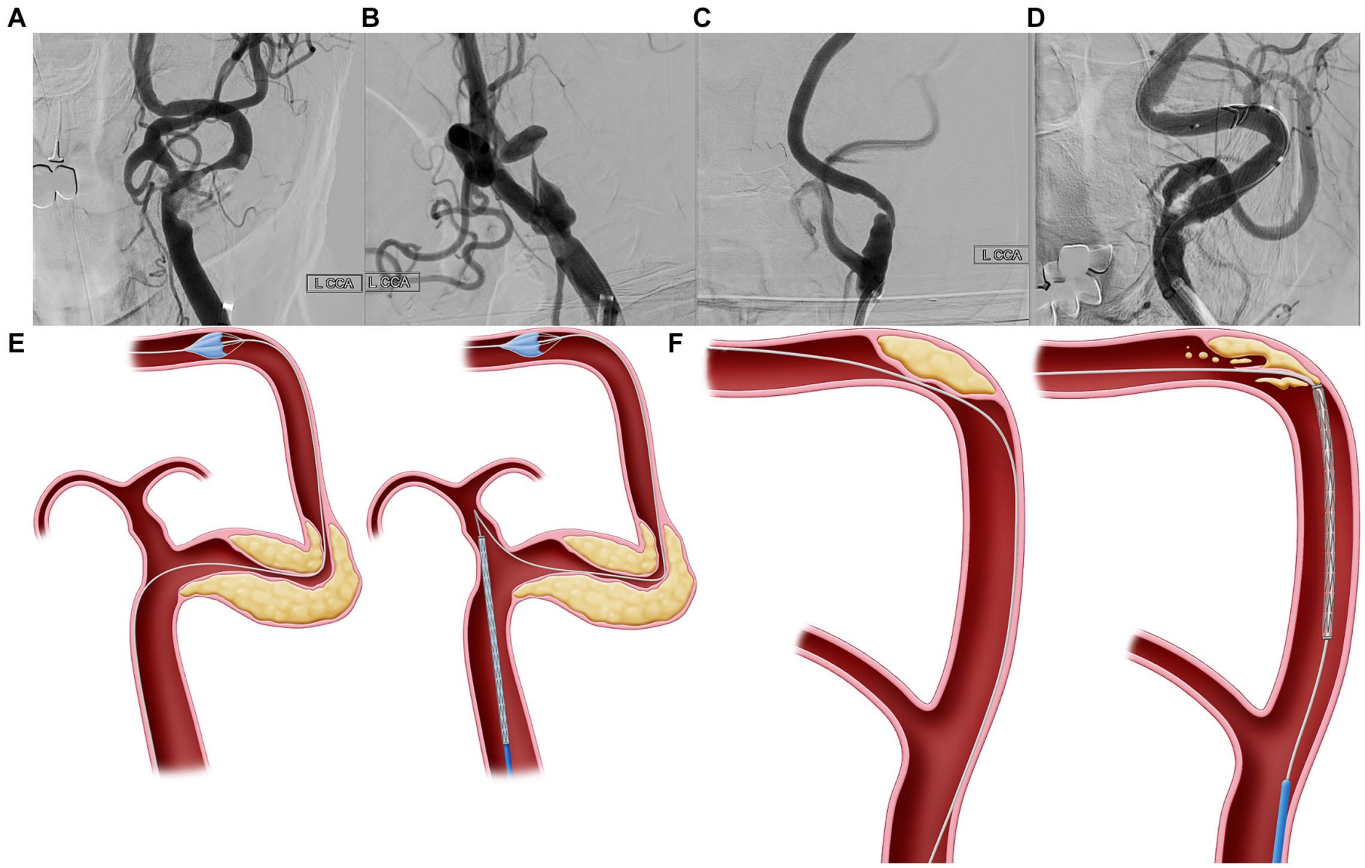
Curve-centered plaque. **(A–D)** Curve-centered plaque (CCP) is a plaque associated with stenosis located within the severe tortuous carotid artery, which is the lesion target of the stenting procedure. **(E)** In vascular structures with severe curves, when delivering the device through a wire which has advanced to the distal segment, the devices with wire may fall into the external carotid artery due to failure to overcome the angle. Thus, since the delivery of devices is impossible, there is no choice but to advance the guiding system to the stenotic area. In this case, more than necessary power is accumulated in the guiding catheter during device delivery, which can possibly lead to dangerous or uncontrolled events. **(F)** Second, because the plaque is located at the center of the bent carotid artery, the CAS device cannot be advanced along the wire and can possibly dig into the plaque. If the interventionist pushed more of the device to pass through the bent vessel, the wire would adhere more closely to the center of the curve where the plaque was located, potentially increasing the risk of periprocedural complications.

The anatomic characteristics of the lesions were analyzed individually via neuroimaging (MRA, CTA, DUS, and DSA) before the procedure. Neuroimaging was performed to assess the lesion shape, location, ulceration, calcification, degree of stenosis, lesion length, and contralateral carotid artery status. Using the DUS B-mode test, plaque characteristics were classified as hypoechoic, isoechoic, or hyperechoic. The ICA tortuosity index (TI) was calculated for all the available angiograms. The ICA TI was determined based on a 3-point system that measured the degree of deviation of the internal carotid from the axis between the common and distal internal carotid arteries. The TI was measured to determine the morphology of the segment between the initiation of the ICA and the sites of the EPDs. The TI was defined as the sum of all angles diverging from the ideal straight axis. Angle *α* was defined as the angle between the initial ICA’s axis and the ICA’s tangent. Angle *β* was defined as the angle between the axes of the first and second segments of the ICA. The sum of angle *α* and β was defined as TI (10). CCP was defined as a large plaque associated with stenosis located within the severely tortuous carotid artery and was the lesion target of the stenting procedure (Figures 2A–D). The ostial-centered lesion was defined as the location of maximal stenosis in the internal carotid ostium (11). Three neurointerventionists retrospectively evaluated all DSA images for the following anatomical higher-risk appearances: high ICA TI, CCP, degree of carotid stenosis, lesion location (carotid bulb or supra-bulb), ostial-centered lesion, length of stenosis >15 mm, and ulceration and calcification. Additionally, the echogenicity (hypoechoic, isoechoic, or hyperechoic) of the plaque on DUS was evaluated.

### Outcome evaluations

Stroke neurologists re-evaluated the neurological status after the procedure, and the patients were monitored in the neurointensive care unit for 1 day. All patients were evaluated with simple diffusion-weighted images immediately after the procedure. Patients with a >2-point increase in the National Institutes of Health Stroke Scale (NIHSS) were evaluated using non-contrast brain CT or MRI. DUS were performed to evaluate the restenosis at discharge. Clinical outcomes were assessed by measuring mRS scores at 90 days. Other clinical and imaging outcomes evaluated included early neurological deterioration (END), length of hospital stay, re-stenosis on follow-up imaging, and periprocedural complications. DUS was performed before the procedure and at 1, 6, and 18 months after the intervention. Procedure time was defined as the interval from the puncture to the final angiography.

### Periprocedural complications

Periprocedural complications were defined as newly developed neurological deterioration or abnormal neuroimaging features that developed within 1 week after CAS. We regarded any neurological symptom, including ischemic stroke, a malpositioned stent, vasospasm, dissection, myocardial infarction, distal occlusion, an asymptomatic DWI lesion, hyperperfusion/reperfusion events, postprocedural headache, ocular symptoms, instant thrombosis, pseudoaneurysm, and any cardiorespiratory arrest after the procedure as complications of CAS. Cardiorespiratory arrest was defined as a new condition requiring endotracheal intubation or chest compression within 1 day after CAS.

In addition to those mentioned above, complications associated with CAS include access site complications such as hematoma and infection, and contrast medium-induced nephropathy.

### Statistical analysis

We compared baseline characteristics, clinical status, procedural characteristics, and clinical outcomes between the patients who experienced complications and those who did not. Differences in baseline categorical variables were compared using Pearson’s chi-square test or Fisher’s exact test. In contrast, differences in continuous variables were compared using Student’s *t*-test or the Mann–Whitney *U*-test, as appropriate. Multivariate logistic regression analysis was performed to identify the independent variables contributing to complications. Variables with a *p-*value <0.2 in a univariate analysis were included as candidate variables in a multivariate analysis and removed by backward stepwise selection. Additional forward selection analyses confirmed the final model. Statistical significance was defined as a two-tailed *p*-value of <0.05. All statistical analyses were performed using the R statistical software (version 2.14.0; R Foundation for Statistical Computing, Vienna, Austria).

## Results

Over 10 years, 218 carotid stenting procedures were performed, of which 148 met the inclusion criteria (Figure 1). A total of 148 patients (64 women [43.2%]; median age, 73.0 [interquartile range, 65.5–79.0]) were analyzed (Table 1). There were no differences between the two groups regarding age, sex, the baseline mRS score, or underlying disease. However, the complications group had a higher initial NIHSS score than did the non-complications group, and the proportion of patients with symptomatic stenosis was higher in the complications group than in the non-complications group.

**Table 1:** Baseline characteristics.

Any complications	All (*N* = 148)	No complication (*N* = 109)	Complication (*N* = 39)	*p*
Age, years	73.0 (65.5–79.0)	72.0 (65.0–78.0)	76.0 (67.5–81.5)	0.26
Sex, male	84 (56.8%)	62 (56.9%)	22 (56.4%)	0.99
Age > 80 years	34 (23.0%)	22 (20.2%)	12 (30.8%)	0.26
Initial NIHSS	0.0 (0.0–2.5)	0.0 (0.0–1.0)	1.0 (0.0–4.0)	0.01
Symptomatic stenosis	79 (53.4%)	51 (46.8%)	28 (71.8%)	0.01
Baseline mRS				0.06
0	77 (52.0%)	64 (58.7%)	13 (33.3%)	
1	38 (25.7%)	24 (22.0%)	14 (35.9%)	
2	14 (9.5%)	9 (8.3%)	5 (12.8%)	
3	19 (12.9%)	12 (11.0%)	7 (17.9%)	
Lesion side (Left)	70 (47.3%)	48 (44.0%)	22 (56.4%)	0.25
Hypertension	122 (82.4%)	94 (86.2%)	28 (71.8%)	0.07
Diabetes mellitus	51 (34.5%)	38 (34.9%)	13 (33.3%)	0.99
Hypercholesterolemia	69 (46.6%)	49 (45.0%)	20 (51.3%)	0.62
Previous stroke	40 (27.0%)	29 (26.6%)	11 (28.2%)	0.99
Coronary artery disease	36 (24.3%)	27 (24.8%)	9 (23.1%)	0.99
Smoking	51 (34.5%)	38 (34.9%)	13 (33.3%)	0.99
Alcohol consumption	42 (28.4%)	30 (27.5%)	12 (30.8%)	0.86
HbA1c	6.1 (5.8–6.6)	6.2 (5.8–6.7)	6.0 (5.5–6.5)	0.09
Syncope like episode	9 (6.1%)	7 (6.4%)	2 (5.1%)	0.99

NIHSS, National Institutes of Health Stroke Scale; mRS, modified Rankin scale. HbA1c, hemoglobin A1c.

There was no difference between the two groups concerning the devices used, ICA TI, degree of stenosis, ulceration, echogenicity, or contralateral carotid artery occlusion; however, the frequency of CCP was significantly higher in the complication group than in the non-complication group (Table 2).

**Table 2:** Characteristics of procedures and carotid artery stenosis.

Any complications	All (*N* = 148)	No complication (*N* = 109)	Complication (*N* = 39)	*P*
Devices				
Open cell type stent	124 (83.8%)	88 (80.7%)	36 (92.3%)	0.375
Pre-stent balloon angioplasty	147 (99%)	108 (99.1%)	39 (100%)	0.346
Post-stent balloon angioplasty	119 (80.4%)	89 (81.6%)	30 (76.9%)	0.309
Embolic protection device	148 (100%)	109 (100%)	39 (100%)	0.192
Concentric type	131 (88.5%)	96 (88.1%)	35 (89.7%)	
Eccentric type	13 (8.8%)	10 (9.1%)	3 (7.7%)	
Mo.Ma Ultra™	4 (2.7%)	3 (2.8%)	1 (2.6%)	
Carotid artery characteristics				
ICA tortuosity index	180.9 (166.6–198.2)	181.4 (168.0–198.0)	177.6 (166.5–198.1)	0.78
CCP	58 (39.2%)	36 (33.0%)	22 (56.4%)	0.02
Location of stenosis (bulb)	91 (61.5%)	67 (61.5%)	24 (61.5%)	0.99
Degree of stenosis	77.0 (68.0–90.0)	75.0 (67.0–90.0)	80.0 (70.5–90.0)	0.17
Ostial centered lesion	35 (23.6%)	25 (22.9%)	10 (25.6%)	0.90
Length of stenosis >15 mm	65 (43.9%)	49 (45.0%)	16 (41.0%)	0.81
Ulceration	72 (48.6%)	51 (46.8%)	21 (53.8%)	0.57
Calcification	69 (46.6%)	53 (48.6%)	16 (41.0%)	0.53
Echogenicity				0.49
Hypoechoic	32 (21.6%)	21 (19.3%)	11 (28.2%)	
Isoechoic	55 (37.2%)	41 (37.6%)	14 (35.9%)	
Hyperechoic	61 (41.2%)	47 (43.1%)	14 (35.9%)	
Contralateral carotid artery stenosis >50%	44 (29.7%)	33 (38.3%)	11 (28.2%)	0.82
Contralateral carotid artery occlusion	6 (4.1%)	5 (4.6%)	1 (2.6%)	0.94

CCP, curve-centered plaque.

Complications occurred in 39 (26.3%) of 148 patients, most of which were minor and transient. Asymptomatic DWI high-signal lesions were the most common complication, occurring in 12 (30.8%) patients. Ischemic stroke occurred in six (15.4%) patients, but all had minor symptoms, and no patients had sequelae after 3 months.

When comparing the clinical outcomes between the two groups, there were no significant differences in the prevalence of re-stenosis, mRS at 90 days, or mortality at 90 days. However, the procedure and hospitalization periods were longer in the complications group than in the non-complications group (Table 3).

**Table 3:** Clinical outcome in patients who underwent carotid stenting.

Any complications	All (*N* = 148)	No complication (*N* = 109)	Complication (*N* = 39)	*P*
Puncture to final angiography time, min	44.5 (35.0–61.5)	42.0 (34.0–58.0)	49.0 (38.0–69.0)	0.04
Hospital length of stay, days	8.0 (6.0–10.0)	7.0 (5.0–12.0)	10.0 (7.0–14.0)	0.04
Restenosis on 6 months	11 (7.4%)	8 (7.3%)	3 (7.7%)	0.99
mRS at 90 days				0.06
0	77 (52.0%)	64 (58.7%)	13 (33.3%)	
1	38 (25.7%)	24 (22.0%)	14 (35.9%)	
2	14 (9.5%)	9 (8.3%)	5 (12.8%)	
3	19 (12.8%)	12 (11.0%)	7 (17.9%)	
Mortality at 90 days	0 (0.0%)	0 (0.0%)	0 (0.0%)	

mRS, modified Rankin scale.

The univariate analysis revealed that symptomatic stenosis, CCP, and procedure time were associated with periprocedural complications. However, in the multivariate analysis, only CCP was an independent risk factor for periprocedural complications of CAS (Table 4).

**Table 4:** Factors associated with any complications of carotid stenting.

	Univariate analysis	*P*	Multivariate analysis	*P*
Age > 80 years	1.76 (0.76–3.98)	0.18		
Symptomatic stenosis	2.89 (1.34–6.62)	0.01	2.29 (1.01–5.41)	0.05
Initial NIHSS	1.09 (1.00–1.19)	0.04		
CCP	2.62 (1.25–5.61)	0.01	2.23 (1.02–4.88)	0.04
TI	0.78(0.54–1.02)	0.317		
Symptom to procedure time	0.99 (0.95–1.03)	0.77		
Carotid bulb stenosis or Suprabulb	1.16 (0.48–2.65)	0.73		
Puncture to final angiography time, min	1.02 (1.00–1.04)	0.02	1.01 (1.00–1.03)	0.14
Length of stenosis >15 mm	0.85 (0.40–1.78)	0.67		
Ostial centered lesion	1.16 (0.48–2.65)	0.73		

Univariate results are reported as odds ratios (95% CI), which multivariate results are reported as Adjusted odds ratios (95% CI). CI, confidence interval; NIHSS, National Institutes of Health Stroke Scale; CCP, curve-centered plaque. TI, Tortuosity Index.

## Discussion

A CCP is a large plaque associated with stenosis located within the severely tortuous carotid artery and is the lesion target of the stenting procedure. Delivering the CAS system through a CCP to avoid plaque irritation during the procedure is challenging. Our study showed that complications occurred more frequently in cases with CCP at the proximal ICA. Thus, we suggest a more specific concept of ICA tortuosity to predict periprocedural complications during CAS.

The CAS is currently performed as an alternative interventional treatment option for CEA (12). It is an effective and safe procedure for increasing blood flow through a previously blocked artery and reducing the risk of stroke without requiring general anesthesia or open surgery (13, 14). However, preprocedural considerations and anatomical risk factor analyses for CAS are currently insufficient. We investigated the risk factors that predict periprocedural complications from the standpoint of neurointerventionists. In our study, periprocedural complications occurred in 26.3% (39/148) of the CAS procedures performed over 10 years. The incidence of complications was relatively frequent, probably because the researchers included complications that were minor or could be ignored. Thus, it rarely lasted for 90 days, or it caused severe neurological deficits.

The CAS is non-inferior to CEA regarding the efficacy and safety of carotid revascularization (3, 4, 15). It offers the advantages of simpler and shorter procedure than that of CEA (13). Nevertheless, minor and non-disabling stroke incidence is more frequently reported in patients who undergo CAS than in those who undergo CEA, especially during the perioperative period (3, 4, 12, 16). Long-term procedure-related deaths or strokes are more frequent in the CAS group than in the CEA group, although the differences are minor (4). There have been several studies on the impact of extracranial ICA tortuosity on the clinical outcome of CAS (8, 10). However, screening patients for CAS according to ICA tortuosity does not provide clear guidelines or distinctive features in clinical practice.

Specifically, two major problems occur during CAS in the carotid artery with CCP. In vascular structures with severe curves, when delivering the device through a wire that has advanced to the distal segment, the devices with the wire may fall into the external carotid artery due to failure to overcome the angle (Figure 2E). Thus, because the delivery of devices is impossible, the guiding system must be advanced toward the stenotic area. In this case, more than necessary power accumulates in the guiding catheter during device delivery, leading to dangerous or uncontrolled events. Second, because the plaque is located at the center of the bent carotid artery, CAS devices cannot be advanced along the wire and can dig into the plaque (Figure 2F). If the interventionist pushed more of the device to pass through the bent vessel, the wire would adhere more closely to the center of the curve where the plaque was located, potentially increasing the risk of periprocedural complications.

The present study had several limitations. First, the inherent biases owing to the single-center retrospective design, with a relatively small number of patients in each group, affected the results. Second, the definition of CCP is still non-specific and vague because the precise angle of the carotid artery was not determined. Nevertheless, CCP is a more intuitive and practical tool for screening high-risk CAS groups. Third, we only assessed the tortuosity of the extracranial ICA; the tortuosities of other relevant vessels, including the aortic arch and common carotid artery, were not assessed. In addition, we assessed the vessel anatomy based on anteroposterior and lateral projection without three-dimensional imaging, which can provide the most precise angle measurements. However, our study was designed to pursue a practical situation without advanced neuroimaging. Above all, the 10-year study period may affected the results, associated with developing medications and devices. Nevertheless, no significant novel therapeutic advances have been made in the endovascular treatment of carotid artery stenosis for about 10 years.

Furthermore, clinical outcomes are affected by other factors, including the skill or experience level of the neurointerventionist and the type of device selected. Therefore, further studies with larger sample sizes are warranted. Nevertheless, in patients with CCP, it might be better to consider CEA rather than CAS, and developing devices and techniques to overcome CCP is necessary.

## Conclusion

In conclusion, although minor and transient, CCP was independently associated with a high frequency of periprocedural complications during CAS. Careful patient screening is crucial for improving the outcomes of CAS.

## Data Availability

The raw data supporting the conclusions of this article will be made available by the authors, without undue reservation.
